# Three Valuable Peptides from Bee and Wasp Venoms for Therapeutic and Biotechnological Use: Melittin, Apamin and Mastoparan

**DOI:** 10.3390/toxins7041126

**Published:** 2015-04-01

**Authors:** Miguel Moreno, Ernest Giralt

**Affiliations:** Chemistry and Molecular Pharmacology, Institute for Research in Biomedicine (IRB Barcelona), Baldiri i Reixac, 10, Barcelona 08028, Spain

**Keywords:** bee, wasp, venom, melittin, apamin, mastoparan

## Abstract

While knowledge of the composition and mode of action of bee and wasp venoms dates back 50 years, the therapeutic value of these toxins remains relatively unexploded. The properties of these venoms are now being studied with the aim to design and develop new therapeutic drugs. Far from evaluating the extensive number of monographs, journals and books related to bee and wasp venoms and the therapeutic effect of these toxins in numerous diseases, the following review focuses on the three most characterized peptides, namely melittin, apamin, and mastoparan. Here, we update information related to these compounds from the perspective of applied science and discuss their potential therapeutic and biotechnological applications in biomedicine.

## 1. Introduction

The order of *Hymenoptera* divides into two suborders *Symphyta* and *Apocrita*. This latter represents the first evolutionary step in the development of the hymenopteran venom system [1]. Furthermore, the suborder *Apocrita* is traditionally divided into two groups, the *Aculeata* and Parasitica. At the same time, *Aculeata* contains several superfamilies, *Vespoidea* and *Apoidea* among others. Inside Vespoidea is the family *Vespidae*, which represents a large and diverse family of cosmopolitan wasps as does the family *Apidae*, inside the family *Apoidea*, which comprises many species of bee, among them the common honey bee. The sting of members of the *Aculeata* group is modified for injecting venom into prey or predators. The chemical composition of these insect venoms is complex, encompassing, a mixture of many kinds of compounds, proteins, peptides, enzymes, and other smaller molecules. This mixture of biologically active substances can exert toxic effects, contributing to certain clinical signs and symptoms of envenomation. Human responses to stings include pain, small edema, redness, extensive local swelling, anaphylaxis, and systemic toxic reaction [2]. However, several venom components have been widely used in Oriental medicine to relieve pain and to treat inflammatory diseases such as rheumatoid arthritis and tendonitis. Other potential venom-related treatments for immune-related diseases, infections, and tumor therapies are currently under investigation. In this review, we focus our attention on the most recent and innovative therapeutic and biological applications of three of the most widely known components of bee and wasp venom, namely melittin, apamin and mastoparan (see Table 1). Melittin and apamin are the only found in the genus *Apis*. However, mastoparan is found in more genera, such as *Vespa*, *Parapolybia*, *Protonectarina*, *Polistes* and *Protopolybia*.

**Table 1:** Protein primary structure of melittin, apamin and mastoparan.

Melittin	GIGAVLKVLTTGLPALISWIKRKRQQ
Apamin	C_1_NC_2_KAPETALC_1_ARRC_2_QQH *
Mastoparan	INLKALAALAKKIL

* The cysteines’ subscripts of Apamin sequence represent the disulfide bridges between Cys in positions 1 with 11, and Cys in positions 3 with 15.

Hymenoptera venom therapy, in particular that involving bee venom (apitoxin), was practiced in ancient Egypt, Greece, and China and, improved by modern studies of apitherapy during the 19th century. However, precise knowledge of the composition and mode of action of such venom dates back only 50 years. The advent of electrophoresis, chromatography and gel-filtration, together with pharmacological and biochemical techniques, brought about the identification of a number of components of bee and wasp venoms. Later on, improved and novel techniques of purification and sequence analysis by Edman degradation and the new analytical chemistry technique mass spectrometry (MS) allowed accurate characterization of the major components in venoms. On the other hand, the advance in transcriptomic and genomic analysis also have helped to identify genes expressed in venom glands. The amount of venom protein released in a sting varies between species, ranging between 50 and 140 micrograms for bees [3,4] and between 1.5 and 20 micrograms for wasps [3,5]. Proteins and peptides comprise the main components of the venoms of these insects (see Table 2). The venoms also contain volatile alarm pheromones (4%–8%), such as iso-pentyl acetate, 2-nonanol, and n-butyl acetate, which trigger defensive responses from nearby insects [6]. Bee and wasp venom share several biologically active proteins and neurotransmitters, such as phospholipases A_2_ and B, hyaluronidase, serotonin, histamine, dopamine, noradrenaline and adrenaline. However, some peptides are exclusive to each insect, namely melittin, apamin and mast cell degranulating (MCD) peptide to bees, and mastoparan and bradykinin to wasps.

**Table 2:** Main proteins and peptides found in bee and wasp venom.

Bee venom	Wasp venom	Type and MW (Da)	% Compound *	Toxic **
Phospholipase A_2_	Phospholipase A_2_	Enzyme (~18 kDa)	10–12	Yes
Phospholipase B	Phospholipase B	Enzyme (~26 kDa)	1	Yes
Hyaluronidase	Hyaluronidase	Enzyme (~54 kDa)	1.5–2	Yes
Phosphatase	Phosphatase	Enzyme (~60 kDa)	1	No
α-Glucosidase	α-Glucosidase	Enzyme (~170 kDa)	0.6	No
Melittin	-	Peptide (2847 Da)	40–50	Yes
Apamin	-	Peptide (2027 Da)	2–3	Yes
MCD peptide	-	Peptide (2593 Da)	2–3	Yes
-	Mastoparan	Peptide (1422 Da)	No data	Yes
-	Bradykinin	Peptide (1060 Da)	No data	No

* The percentages of compounds correspond to the venom itself and do not take into account the water content. Concentration can differ between bee and wasp species. ** This toxicity refers to the potential toxicity that each component could have. It is based on the cytotoxic and immunologic effect of each protein described in the text.

### 1.1. Enzymes

Focusing on enzymes related to toxicity, phospholipase and hyaluronidase are the two major enzymatic proteins present in hymenoptera venom. These enzymes can trigger an immune response, inducing IgE response in susceptible individuals [7].

Phospholipase A_2_ (PLA_2_) is a calcium-dependent enzyme that hydrolyzes the sn-2 ester of glycerophospholipids to produce a fatty acid and a lysophospholipid. It destroys phospholipids, disrupting the integrity of the lipid bilayers, thus making cells susceptible to further degradation. In fact, PLA_2_ reaction products, such as lysophosphatidylcholine, lysophosphatidic acid and sphingosine 1-phosphate, can have cytotoxic or immunostimulatory effect on diverse cell types, causing inflammation and immune responses [8].

Phospholipase B (PLB), also known as lysophospholipase, is an enzyme found in very low concentrations in some venoms. With the capacity to cleave acyl chains from both sn-1 and sn-2 positions of a phospholipid, PLB shows a combination of PLA_1_ and PLA_2_ activities [9]. 

Hyaluronidase is commonly known as a “spreading factor” because it hydrolyzes the viscous polymer hyaluronic acid into non-viscous fragments. When extracellular matrix is destroyed by hyaluronidase, the gaps between cells facilitate the invasion of venom toxins. Therefore, venom penetrates tissues and enters blood vessels, thus catalysing systemic poisoning. Furthermore, hydrolyzed hyaluronan fragments are pro-inflammatory, pro-angiogenic and immunostimulatory, thus inducing faster systemic envenomation [10].

### 1.2. Peptides

Mast cell degranulating (MCD) peptide is a cationic peptide with 22 amino acid residues that has a similar structure to apamin, being cross-linked by two disulphide bonds [11]. This peptide is a potent anti-inflammatory agent; however, at low concentration it is a strong mediator of mast cell degranulation and histamine release from mast cells, which are present in the blood supply and in all tissues perfused by blood [12].

Bradykinin is a physiologically active peptide that belongs to the kinin group of proteins. Bradykinin and related kinins act on two receptors, designated as B1 and B2. The former is expressed only as a result of tissue injury and it is thought to play a role in chronic pain. In contrast, the B2 receptor is constitutively expressed, participating in vasodilatation via the release of prostacyclin, nitric oxide, and endothelium-derived hyperpolarizing factor, thus contributing to lowering blood pressure [13].

Adolapin is a peptide that was first isolated from bee venom in the 80s. It exerts a potent analgesic effect and anti-inflammatory activity in rats, blocking prostaglandin [14]. Tertiapin, also from bee venom, is a 21 amino acid peptide that blocks certain types of inwardly rectifying potassium channels [15]. The peptides Scapin, Scapin-1, and Scapin-2 are all 25 amino acid residues in length and share a similar secondary structure, with a disulfide bridge between Cys 9 and Cys 20. These peptides have been isolated from the venom of various species, such as Scapin from European *Apis mellifera* [16], Scapin-1 from Chinese *Apis mellifera* [17], and Scapin-2 from the Africanized honeybee. These compounds induce leukotriene-mediated hyperalgesia and edema [18]. Melittin F contains 19 amino acid residues and differs from melittin in that the first seven residues of the *N*-terminus are absent, therefore it resembles a fragment of melittin [19]. Cardiopep is a peptide isolated from whole bee venom that has beta adrenergic and anti-arrhythmic effects [20]. Antigen 5, one of the major allergens in all wasp venoms, has an unknown biological function [21]. Other recently isolated and featured peptides show antimicrobial activity, playing a key role in preventing potential infection by microorganism during prey consumption by insect larvae. Examples of such peptides include Anoplin from *Anoplius smariensis* [22], Crabrolin from *Vespa crabro* [23], Decoralin from *Oreumene decoratus* [24], Eumentin from *Eumenes rubronotatus* [25], Melectin from *Melecta albifrons* [26], and Protonectin from *Agelaia pallipe pallipes* [27].

### 1.3. Low Molecular Weight Compounds

Bee and wasp venoms also contain small molecules, such as minerals, amino acids, and physiologically active amines, such as catecholamines. Among this category, histamine is one of the major components. This organic nitrogenous compound participates in the inflammatory response by increasing the permeability of capillaries. In a similar manner, the catecholamines dopamine and nor-adrenaline increase heartbeat, thereby enhancing venom circulation and thus, its distribution [28]. However, like histamine, the effects of these two catecholamines are largely overshadowed by those of other components of venom. Serotonin can act as an irritant and can contribute to the pain caused by the venom. Finally, high levels of acetylcholine are detected only in wasp venom. Acetylcholine can increase perceived pain of a sting by stimulating pain receptors synergically with histamine effects.

## 2. Bee Venom (Apitoxin)

Since the first studies in apitherapy at the beginning of the 20th century, multiple therapeutic applications for bee venom have been developed for certain diseases. However, although we have a better understanding of the mechanisms of action of bee venom components, many questions remain unanswered. Given the anti-inflammatory properties of this venom, various forms of traditional bee venom therapy, including the administration of live stings, injection of venom, and venom acupuncture have been used to relieve pain and to treat chronic inflammatory diseases such as rheumatoid arthritis and multiple sclerosis [29,30]. This traditional medicine also has been used for other diseases like cancer [29], skin conditions [31], and recently even for Parkinson’s disease [32]. In addition, Apitox^®^ (Apimeds, Inc., Seongnam-si, Korea), purified bee venom from *Apis mellifera*, is an FDA-approved subcutaneous injectable product for relieving pain and swelling associated with rheumatoid arthritis, tendinitis, bursitis and multiple sclerosis [33]. From a scientific perspective, special mention is given to two particular reviews [34,35] that assessed the evidence of a non-systematic manner of designing, performing, and analyzing clinical studies of bee venom acupuncture for rheumatoid arthritis and musculoskeletal pain. After evaluating the safety and efficacy of these studies, the results showed evidence of effectiveness. However, the authors highlighted that not only was the total human sample size too small, but the quality of experimental design was varied and sometimes inadequate. Recently, two pilot studies addressing chronic pain of the neck and lower back have were designed under a rigorous randomized clinical trial with the aim to evaluate the true effect of bee venom acupuncture [36,37]. Therefore, new protocolized studies are in the pipeline to validate the efficiency of this novel therapy. Furthermore, purified and synthesized bee venom components and their derivatives have led to novel pharmaceutical agents. In the following sections, we address in more detail the applications of two of the most studied peptides obtained from bee venom, namely melittin and apamin.

### 2.1. Therapeutic and Biotechnological Applications of Melittin

Melittin, the main component of bee venom, hyaluronidase and PLA_2_ are the three major causes of allergic reactions to this venom [38]. An amphiphilic peptide comprising 26 amino acid residues, and in which the amino-terminal region is predominantly hydrophobic and, the carboxyl-terminal region is hydrophilic. Melittin is the principal active component of apitoxin and is responsible for breaking up and killing cells. When several melittin peptides accumulate in the cell membrane, phospholipid packing is severely disrupted, thus leading to cell lysis [39]. Melittin triggers not only the lysis of a wide range of plasmatic membranes but also of intracellular ones such as those found in mitochondria. PLA_2_ and melittin act synergistically, breaking up membranes of susceptible cells and enhancing their cytotoxic effect [40]. This cell damage, in turn, may lead to the release of other harmful compounds, such as lysosomal enzymes from leukocytes, serotonin from thrombocytes, and histamine from mast cells, which can all lead to pain.

Although melittin is the most studied and known bee venom peptide, its development for clinical applications remains mainly in preclinical phases. At the moment of writing, no products for human use are available on the market. Some patents and promising studies have focused on bacterial and viral infections, immunologic adjuvants, rheumatoid arthritis, arteriosclerosis, cancer, and endosomolytic properties for drug delivery.

#### 2.1.1. Antimicrobial Properties of Melittin for Therapeutic Use

Antimicrobial peptides (AMPs) have been widely studied as an alternative to conventional antibiotics, especially for the treatment of drug-resistant infections [41]. Hundreds of AMPs have been isolated, and several thousand have been *de novo* designed and synthesized. Despite displaying extensive sequence heterogeneity, most of these peptides share two functionally important features, namely a net positive charge and the ability to adopt an amphipathic structure. Melittin is considered to show strong antimicrobial properties and it also has hemolytic activity and marked allergenic properties. Early studies using individual peptide analogs of melittin showed that the initial step of the mechanism underlying the hemolytic and antimicrobial activity of this venom peptide involves interactions with the lipid groups of the membrane [42]. The structural requirements for the action of melittin, its orientation, aggregation state, current view of pore formation, and also its various cellular actions are discussed in detail in an excellent review by Dempsey [43]. Bruce Merrifield performed pioneering work on improving the features of antimicrobial peptides, shortening their sequences and increasing their activity. In particular, a hybrid undecapeptide derived from the well-known cecropin A and melittin was found to be sufficient for antifungal and antibacterial activities, while displaying low cytotoxicity [44]. This hybrid version was later improved with retro and retroenantio analogs [45]. Indeed, a patent of several active d-peptides with antibiotic and antimalarial activity was even filed [46]. Despite the therapeutic efficacy of antimicrobial peptides, their use is limited due to poor *in vivo* bioavailability caused by instability, cytotoxicity, hydrophobicity, in addition, the cost production is an issue [47]. In parallel to antimicrobial peptides for therapeutic use in humans, these peptides can be applied to fight economically important plant pathogens, which are currently one of the major factors limiting crop production worldwide [48]. A library of linear undecapeptides derived from cecropin-melittin hybrids have been tested against phytopathogenic bacteria and patented for future use in phytosanitary compositions [49]. In this regard, a promising peptide called BP76 has been identified for this purpose [50].

#### 2.1.2. Antimicrobial Properties of Melittin for Biotechnological Use

The idea of using antimicrobial peptides has also been translated to coatings for medical devices. Currently, a number of companies are turning their attention to the use of antimicrobial coatings of cationic peptides, such as melittin, for contact lenses in order to prevent the growth of undesirable microorganisms. Contact lenses made of materials comprising hydrogels and antimicrobial ceramics that contain at least one metal (selected from Ag, Cu and Zn) are available. However, although these polymeric compositions do have antimicrobial properties, they do not have all the properties desired for extended-wear contact lenses. Antimicrobial coatings containing covalently bound antimicrobial peptides exhibit diminished activity when compared that of the unbound corresponding antimicrobial peptides in solution. To overcome these drawbacks, Novartis has patented a method to produce contact lenses with an antimicrobial metal-containing layer-by-layer (LbL) [51]. In its LbL design, at least one layer has a negatively charged polyionic material, having -COO-Ag groups or silver nanoparticles.

#### 2.1.3. Anti-Viral Properties of Melittin for Therapeutic Use

The antiviral activities described for melittin and its analogs are caused by specific intracellular events, with the selective reduction of the biosynthesis of some viral proteins, as reported for the melittin analog Hecate on herpes virus-1 [52], and for melittin itself on HIV-1-infected lymphoma cells [53]. In the 90s, active melittin was presented to provide an improved composition complementary to azidothymidine (AZT) to inhibit the reverse transcriptase and growth of HIV-infected cells [54]. Recently, a similar idea has been patented, whereby melittin is carried in a nanoparticle construct designed to be used as a topical vaginal virucide [55,56].

#### 2.1.4. Vaccines

Related to fields of immunology and vaccinology, the 90s also witnessed great progress in therapeutic approaches based on vaccination against infectious pathogens. Despite these advances in the identification of new antigens and their immunological mechanisms, the immune response in most cases continues to be very weak. Therefore, to improve the response, effective adjuvants to enhance the immunogenicity of target antigens must be used. A few years ago, Rinaldo Zurbriggen presented a novel adjuvant system based on melittin and analogs capable of eliciting strong immune responses against target antigens, thus reducing the risk of toxic side effects associated with the use of adjuvants [57].

#### 2.1.5. Inflammatory and Rheumatic Applications of Melittin

Uncontrolled inflammation can cause extensive tissue damage and is the hallmark of numerous diseases, including rheumatoid arthritis, which results in joint destruction and permanent disability. PLA_2_ is the enzyme responsible for hydrolyzing arachidonic acid from phospholipids, and arachidonic acid is the precursor of eicosanoids, which are thought to mediate inflammation. Melittin and related peptides have been described as anti-inflammatory drugs as they have the capacity to inhibit PLA_2_ [58,59]. However, in this field, melittin competes with a wide variety of non-steroidal drugs, methotrexate, and other biological disease-modifying antirheumatic drugs [60].

#### 2.1.6. Atherosclerosis Applications of Melittin

Atherosclerosis is the major cause of morbidity and mortality worldwide. This specific form of arteriosclerosis is a chronic inflammatory disease of the arteries caused by the accumulation and interaction of white blood cells, remnants of dead cells, cholesterol, and triglycerides on the artery wall. This complex inflammatory process is characterized by the presence of monocytes/macrophages and T lymphocytes in the atheroma, where macrophages secrete pro-inflammatory cytokines, a main cellular component in the development of atherosclerotic plaques [61]. Several *in vitro* studies have shown positive effects of melittin for the treatment of atherosclerosis [62,63]. In addition, *in vivo* experiments have demonstrated the molecular mechanism of the anti-atherosclerotic effects of melittin in mouse models of this disease [64]. This has been the major finding regarding the capacity of melittin to prevent lipopolysaccharide (LPS)/high-fat-induced expression of inflammatory cytokines, proatherogenic proteins, and adhesion molecules.

#### 2.1.7. Cancer Applications of Melittin

Many studies report that melittin inhibits tumor cell growth and induces apoptosis, thereby indicating a potential application of this venom peptide as an alternative or complementary medicine for the treatment of human cancers. A valuable review describing the mechanisms underlying the anticancer effects of melittin has been published [65]. Cells in several types of cancer, such as renal, lung, liver, prostate, bladder, breast, and leukemia, can be targeted by melittin. It is well-known that melittin is a natural detergent with the capacity to form tetramer aggregates on membranes, which lead to disorders in the structure of phospholipid bilayers, changes in membrane potential, aggregation of membrane proteins, as well as the induction of hormone secretion [66]. Furthermore, this membrane disruption directly or indirectly leads to alterations in enzymatic systems, such as G-protein [67], protein kinase C [68], adenylate cyclase [69], and phospholipase A [70]. Melittin can even inhibit calmodulin, a calcium-binding protein that plays a crucial role in cell proliferation [71]. Tumoral cells expose anionic phospholipids, mainly phosphatidylserine, on the external leaflet of the plasma membrane [72], and this feature can allow the preferential binding of cationic peptides, like melittin, relative to normal cells. Melittin studies with numerous types of cancer cells and *in vivo* animal models have demonstrated its antiproliferative activity [73,74]. Furthermore, recent studies have demonstrated that melittin has anti-angiogenesis properties [75,76,77].

However, when a therapeutic dose of melittin is injected *in vivo*, some side effects, such as liver injury and hemolysis, were observed. To minimize these emerging lesions in off-target tissues, the following three strategies have been designed: (1) conjugaton of melittin to an antibody or a targeting component; (2) development of shielded pro-cytolytic melittin systems; and (3) synthesis of melittin-transporting carriers.

With regard to the first approach, a melittin-based recombinant immunotoxin obtained by fusion of genes that encoded an antibody fragment derived from the murine monoclonal antibody K121 with an oligonucleotide encoding melittin was tested successfully *in vitro* [78]. Another study was based on a recombinant immunotoxin of melittin fused to an anti-asialoglycoprotein receptor (ASGPR) single-chain variable fragment antibody (Ca) which conferred targeting and ASGPR-specific cytotoxicity to hepatocellular carcinoma cells [79]. Finally, a recent study characterized a CTLA-4-targeted scFv-melittin fusion protein as a potential immunosuppressive agent for organ transplant. In this regard, the selective cytotoxicity of the peptide construction was confirmed in preliminary biological activity assays [80].

Related to the pro-cytolytic melittin, by taking advantage of tumor matrix metalloproteinase 2 (MMP2) overexpressed on cancer cell membranes, an MMP2 cleavable melittin/avidin conjugate was built. Melittin coupled to avidin becomes inactive, but when released from the conjugate it induces immediate cell lysis [81]. A similar idea was published years later, this time using avadin, the latency-associated peptide (LAP) domain of the transforming growth factor beta (TGF-β). In this approach, LAP dimerization conferred latency to the MMP2-cleavable melittin-LAP fusion protein [82]. 

Regarding pro-cytotoxic melittin systems, a design was based on the mixture of melittin with the anionic detergent sodium dodecyl sulfate formulated into poly(D,L-lactide-co-glycolide acid) nanoparticles by an emulsion solvent diffusion method. The inhibitory *in vitro* effects of these 130 nm-diameter melittin-loaded nanoparticles on breast cancer MCF-7 cells were promising [83]. Another interesting carrier was a pegylated immunoliposome coupled to a humanized antihepatocarcinoma single-chain antibody variable region fragment and loaded with a bee venom peptide fraction [84]. A similar pegylated immunoliposome but using only melittin as cargo and the complete antibody trastuzumab as targeting component was designed to combat HER2-overexpressing human breast cancer cell lines [85]. The three aforementioned nanoparticles are not suitable for systemic administration because melittin can be released in blood vessels during transport, particularly in liposomes, which can be disrupted by the lytic peptide [86]. To overcome this drawback, Samuel A. Wickline’s group developed a perfluorocarbon nanoemulsion vehicle incorporating melittin into its outer lipid monolayer [87]. This nanocarrier of approximately 270 nm in diameter presented favorable pharmacokinetics, accumulating melittin in murine tumors *in vivo* and causing a dramatic reduction in tumor growth without any apparent signs of toxicity [88,89]. Finally, the most recent ultra-small diameter melittin-nanoparticle (<40 nm) successfully tested *in vivo* with few side effects is the patented α-melittin-NP [90,91]. This nanoparticle comprise 1,2 dimyristoyl-sn-glycero-3-phosphatidylcholine (DMPC) decorated with the hybrid peptide formed by peptide D-4F and melittin via a GSG linker, the peptide D-4F being a peptide that mimics a high-density lipoprotein (HDL) [92].

#### 2.1.8. Endosomolytic Properties of Melittin

The strategy of packing and carrying small interference RNA (siRNA) using a wide variety of systems for gene therapy has been increasingly followed in recent years. The efficiency mediated by these drug delivery systems is strongly dependent on their endosomal escape capability, otherwise the siRNA would be degraded in endolysosomes [93]. One mechanism designed for endosomal release is the use of fusogenic peptides, which are generally short amphipathic sequences between 20 and 30 amino acids in length and capable of disrupting biological membranes at endosomal pH [94,95]. One of the first highly innovative studies using melittin consisted of reversibly masking the membrane-active peptide using maleic anhydride derivative [96]. At neutral pH, the lysine residues of melittin were covalently acylated with anhydride, thereby inhibiting the membrane disruption activity of the peptide. Under acidic conditions such as those present within endosomes, the amide bond of the maleamate was cleaved, thus unmasking melittin. Similar studies performed by Ernest Wagner *et al.* showed that melittin analogs with high lytic activity at acid pH enhance the transfection of oligonucleotides in cell cultures and in *in vivo* mouse models [82,97,98,99]. Very recently, a derivative of melittin (p5RHH) was reported to successfully trigger siRNA release into the cellular cytoplasm [100,101]. The company Arrowhead Therapeutics is currently developing ARC-520 as a novel siRNA-based therapeutic to knock down the expression of viral RNAs of chronic hepatitis B virus. They describe the use of a coinjection of a hepatocyte-targeted, *N*-acetylgalactosamine-conjugated melittin-like peptide (NAG-MLP) with a liver-tropic cholesterol-conjugate siRNA (chol-siRNA) targeting coagulation factor VII [102,103]. Preclinical studies with animals as well as Phase I assays have revealed that melittin promotes delivery without generating anti-melittin antibodies. In March 2014, Phase II trials of ARC-520 were started for patients with chronic hepatitis B virus [104].

### 2.2. Therapeutic Applications of Apamin

Apamin is a peptide neurotoxin comprising 18 amino acid residues that is tightly cross-linked by the presence of two disulphide bonds which connect position 1 with 11 and position 3 with 15 [105]. Apamin selectively blocks the small conductance of Ca^2+^-dependent K^+^ channels (SK channels) expressed in the central nervous system (CNS). This type of channel plays a crucial role in repetitive activities in neurons [106], blocking many hyperpolarising-inhibitory effects, including alpha-adrenergic, cholinergic, purinergic, and neurotensin-induced relaxations [107,108].

Since the Hahn and Leditschke’s first descriptions in the 30s of mouse convulsions caused by apamin injection, other symptoms and properties have been ascribed to this rigid octadecapeptide [106]. After intraperitoneal injection in animals, apamin locates not only in the grey matter of the brain but also in the liver and the adrenal cortex [109,110]. Therefore, apamin can no longer be considered an exclusive neurotoxin. Unlike melittin, apamin is a peptide with a highly specific mode of action. It binds and occludes the pore of small conductance Ca^2+^-triggered K^+^ channels (SK), thus acting as an allosteric inhibitor [111] and depressing delayed cellular hyperpolarization. This binding specificity provides apamin electrical properties which have been exploited in biomedical research. Apamin acts mainly on the CNS, where SK channels are widely expressed [112]. SK channels are divided into the following three main classes on the basis of their conductance: (1) large conductance (BK or K1); (2) intermediate conductance (IK or K2); and (3) small conductance (SK or K3) [113]. These channels, which are activated solely by increases in intracellular Ca^2+^ contribute to regulating the excitability and function of many cell types, including neurons, epithelial cells, T­lymphocytes, and skeletal muscle cells [114]. SK channels are activated by submicromolar concentrations of Ca^2+^, and this activation is mediated by calmodulin [115]. 

#### 2.2.1. Learning Deficit

In excitable cells, the activation of SK channels generates a hyperpolarizing K^+^ current which contributes to the afterhyperpolarisation (AHP) that follows an action potential [116]. This AHP modulates cell firing frequency and spike frequency adaptation, thereby influencing neuronal excitability. SK channels have been implicated in diverse physiological functions such as synaptic enhancement and long­term potentiation. Furthermore, early studies showed that systemic apamin administration facilitates learning and memory. The first such study, using appetitive learning paradigms, reported that systemic apamin injections accelerate acquisition of the bar-pressing response and also accelerate bar-pressing rates [117]. Several studies, listed in Table 3, underscore the relevance of SK channels in information processing and storage at the systems level. Such studies propose that SK channels would be appropriate targets for apamin as a therapeutic treatment for learning deficits.

**Table 3:** Evidences of enhanced learning in animals receiving apamin injections.

Animal studies with apamin	References
Apamin improved rat performance in the novel object recognition task, where habituation of exploratory activity was assessed	[118]
Apamin improved spatial navigation in medial septal-lesioned mice	[119]
Apamin dose-dependently alleviated deficits in spatial reference and working memory induced by partial electrolytic hippocampal lesion	[120]
Apamin attenuated the memory deficits caused by scopolamine, which affect hippocampal and cortical activity	[121]
Apamin-treated mice exhibited fater learning of the platform location during the initial trials in the Morris water maze	[122]
Apamin improved task acquisition in a learned extinction operant behavior protocol	[123]
Apamin enchanced working memory in a medical prefrontal cortex-dependent spatial delayed alternation task	[124]
Apamin facilitated the encoding of contextual fear memory	[125]
Apamin improved performance on the water task in mice with neurofibromatosis 1	[126]

#### 2.2.2. Parkinson’s Disease

Parkinson’s disease (PD) is a neurodegenerative disorder characterized by the progressive loss of dopaminergic (DA) neurons in the subtantia nigra, leading to typical motor symptoms (akinesia, rigidity, rest tremor) [127]. Although therapy with l-Dopa, a precursor of dopamine, has provided benefit for years, the disease progresses slowly, resulting in disability [128]. Recent studies indicate that bee venom [129,130] and apamin [131,132,133] protect DA neurons from degeneration in experimental PD. The use of apamin was patented to overcome the drawbacks of drugs used in the treatment of PD, *i.e.*, l-Dopa [134]. According to this patent, treatment would consist of using from 1–10 micrograms of apamin in a single dose injection. In this approach, apamin would not only protect undamaged neurons but would restore the function of silent neurons. Another recent patent related to the previous one, claims that degenerative brain diseases can be treated with a pharmaceutical composition comprising apamin as an active ingredient and at least one other compound for the treatment of PD and related Parkinsonian disorders [135].

#### 2.2.3. Preserving Red Blood Cells

Whole blood can be stored at 4 °C for three weeks using a CPD (citrate, phosphate, dextrose) anti-coagulant solution. Adding adenine, glucose and/or manitol can prolong blood storage time by two weeks or more. However, there is a necessity to reach a longer period of blood storage. There is a patent that provides methods, compositions, and kits for storing red blood cells for extended periods of time, preventing red blood cell storage lesions, retaining red blood cell deformability, and increasing survival of the cells following transfusion [136]. In some formulas, the composition comprises at least one K^+^ channel blocker agent, including apamin among others.

#### 2.2.4. Blood-Brain Barrier Shuttle

The blood-brain barrier (BBB) is a highly selective part of the neurovascular system that prevents the entry of many substances, including most therapeutics, into the CNS. Paracellular transport between endothelial cells is restricted by tight junctions and transendothelial transport is reduced, thus hindering the use of a high percentage of potential commercialized molecules intended for treatment of the CNS [137]. Several strategies have been implemented to deliver drugs across the BBB, some of which cause structural damage to the barrier by forcibly opening it to allow the uncontrolled passage of drugs. The ideal method for transporting drugs across the BBB should be controlable and should not damage the structure. While a wide range of nanoparticulate delivery systems have been studied with the aim to target therapeutics (low MW drugs, nucleic acids or proteins) to the brain [138], their success rate has been low. The specific distribution of apamin in the CNS and its capacity to cross the BBB [139] make the design of an apamin-based drug delivery system feasible. In fact, a recent study described the promising therapeutic effects of drug-loaded micelles targeted with apamin in reparing spinal cord injury (SCI) in mouse models [140]. However, apamin is neurotoxic at high concentrations, having a relatively low LD_50_ in mice (2.5 micromol/kg) [141]. Bearing this in mind, we assayed a non-toxic analog derived from apamin that bears two ornithine residues instead of arginines residues (ApOO) [142]. We compared and demonstrated the capacity of both peptides to cross the BBB in a cell-based model, thus revealing their potential as BBB carriers [142].

## 3. Wasp Venom

Wasp venom is more variable in composition among species. However, bee and wasp venoms have similar enzymatic composition (see Table 2). A significant difference in the peptide composition of wasp venom is the predominance of mastoparan and bradykinins. Although wasp venom has attracted much less attention than bee venom, extensive research over recent decades has shown the pharmacological properties [143]. In this section, we will focus on the therapeutic applications of the most studied peptide in wasp venom, namely mastoparan.

### 3.1. Therapeutic Applications of Mastoparan

Mastoparan is a membrane-active amphipathic peptide with 14 amino acid residues. It is rich in hydrophobic and basic residues that form amphipathic helical structures, the latter with the capacity to form pores in membranes. Mastoparan induces a potent mitochondrial permeability transition that affects cell viability [144]. The net effect of the mode of action of mastoparan depends on the cell type. In this regard, it causes the secretion of histamine from mast cells, serotonin from platelets, catecholamines from chromaffin cells, and prolactin from the anterior pituitary [145].

Mastoparan exhibits a wide variety of biological effects, including insertion into the membrane bilayer causing membrane destabilization with consequent lysis [146] or direct interaction with G proteins on the cytoplasmatic face, thus perturbing transmembrane signaling [145,147,148,149], stimulation of phospholipases, mobilization of Ca^2+^ from mitochondria and sarcoplasmic reticulum, and cell death by necrosis and/or apoptosis [150,151]. Thus, key biological activities have been described for this peptide, including antimicrobial activity, increased histamine release from mast cells, hemolytic activity [152,153], induction of a potent mitochondrial permeability transition, and tumor cell cytotoxicity. Related to its capacity to induce mitochondrial permeability transition [144], mastoparan has recently been reported to interact with the phospholipid phase of the mitochondrial membrane to induce permeabilization in cyclosporine A-sensitive and insensitive manners but does not interact with any specific receptors or enzymes [154].

#### 3.1.1. Antimicrobial Properties of Mastoparan for Therapeutic Use

Mastoparan alone or in combination with other antibiotics could be a promising alternative for combating multiple-antibiotic resistant bacteria in clinical practice [155]. A number of strategies for optimizing the potency of mastoparan have been addressed, including structural stabilization and charge modification [156,157], achieving synthesis of derivatives and enantiomers [158,159], modulation of hydrophobicity [160,161], and selective acylation/alkylation [162]. However, these studies show that mastoparan activity is gained at the expense of impaired membrane selectivity or *vice versa*, with no distinction between bacterial and mammalian membranes. Thus, the development of new strategies to reduce the toxic side effects of mastoparan, thereby improving the feasibility of clinical applications, are required. However, three independent *in vivo* studies on sepsis, systematic inflammation caused by an infection where bacteria and LPS are potent activators of immune cells, have shown that an analog of mastoparan (mastoparan-1) protects mice from lethal challenge by live bacteria and LPS [163,164,165]. The effects of mastoparan-1 were associated with its bactericidal action and its capacity to neutralize LPS and attenuate inflammatory responses by macrophages.

#### 3.1.2. Anti-Viral Properties of Mastoparan for Therapeutic Use

As for multidrug-resistant bacteria, there is a pressing need to identify novel and broad-spectrum antiviral agents that can be used as therapeutics. A recent study has demonstrated that a mastoparan derivative shows broad-spectrum antiviral activity *in vitro* against five families of enveloped viruses directly via disruption of their lipid envelope structure [166]. However, further studies are needed to demonstrate its therapeutic use.

#### 3.1.3. Cancer Applications of Mastoparan and Mitoparan

As we mentioned previously, mastoparan targets the mitochondrial membrane and causes mitochondrial permeability transition to mediate its tumor cell cytotoxicity. Several studies have demonstrated the antitumor activity of mastoparan and analogs *in vitro* [145,167,168,169]. One potential way to deliver mastoparan and avoid side-effects was presented by Hiyedoshi Harashima *et al.* [170], whereby a transferrin (Tf)-modified liposomes decorated with endosomolytic GALA peptides and, in addition, encapsulating mastoparan, were designed to target the upregulated Tf receptor in tumor cells. Only one *in vivo* study has been carried out, in which mastoparan, administered in a peritumoral way, delayed the subcutaneous development of melanoma in a well-established subcutaneous murine melanoma model and increased survival [171]. It is noteworthy to highlight a potent mastoparan analog, called mitoparan. This peptide shows enhanced amphiphilicity, presenting two additional lysyl side chains in the cationic face and the replacement of a α-aminoisobutiric acid (Aib), a known helix promoter, by an Ala at position 10 [157]. A novel cell-penetranting mitochondriotoxic peptide, mitoparan was modified at its *N*-terminus by adding an RGD motif to confer capacity to selectively bind cell adhesion molecules [172] overexpressed in tumors, which play a significant role in cancer progression and metastasis. This modification would potentially improve the pharmacodynamic parameters of the chimeric mitoparan *in vivo* [168].

Rui Wang *et al.* later patented a new type of acid-activated mitoparan complex by conjugating normal mitoparan with its anionic binding partner via a disulfide linker, where the anionic partner mitoparan, which has three Lys residues replaced by three Glu residues, and two Lys residues by two His residues, shields the cytotoxic activity of mitoparan [173]. Although this chimeric mitoparan complex has been tested only *in vitro*, the designers are optimistic because the complex showed significant enzymatic stability compared with normal mitoparan, thus supporting its potential for *in vivo* application. Recently, we presented a peptide-polymer design strategy to obtain a pro-cytotoxic system based on mitoparan, as cytotoxic peptide, conjugated to a polyglutamic acid polymer through specific cleavage sites that are sensitive to overexpressed tumor proteases, such as MMP-2 and cathepsin B [174]. Our system was also decorated with a specific targeting peptide to HER2^+^ breast tumor cells, thus allowing mitoparan to be released with exquisite spatiotemporal control. It should be noted that the mitoparan that we used for our experiments was the enantiomer form, because normal mitoparan was easily degraded by cellular proteases before its release from the polymer.

#### 3.1.4. Cell-Penetrating Peptide Properties

Cell-penetrating peptides (CPPs) have potential pharmaceutical application in delivering macromolecules into cells. Hundreds of sequences now fall in the CPP classification, and several interesting reviews focusing on internalization mechanism, effect of the cargo, CPP modifications or extensions, protocols and significant effects on penetration capacity have been published [40,105,175,176,177]. Given the capacity of mastoparan and mitoparan to efficiently cross plasma membranes, some researchers have demonstrated the value of these peptides as CPPs [158]. Very recently, the influence of various CCPs on the transport of doxorubicin encapsulating Tf-liposomes across BBB, *in vitro* and *in vivo*, has been addressed [178]. Although mastoparan showed efficient translocation, the other CPPs, namely TAT peptide and penetratin, were more efficient. On the other hand, there is a chimeric galanin-mastoparan peptide called transportan, which contains the first 13 amino acids from the highly conserved amino-terminal part of galanin and the 14 amino acids sequence of mastoparan in the carboxyl terminus [179]. Transportan and some derivatives have the capacity to carry macromolecules [180,181,182].

## 4. Conclusions

Despite the many studies published on bee and wasp venoms, little has been reported on the practical applications of these substances. The main clinical uses of these toxins are based on meridian therapy, focusing on the application of bee venom acupuncture to relevant sites in function of a specific disease or to acupoints. Of note, the single most promising and advanced Phase II trial involving melittin deals with its use as an endosomolytic agent as an effective siRNA delivery system for hepatitis B virus infection [102]. The antimicrobial properties of melittin and mastoparan have been the most studied and developed among the components of wasp and bee venoms. Their mode of action on membranes has drawn attention to AMPs as a universal solution to the growing incidence of drug-resistant infections. However, expectations have not been fulfilled as quickly as initially imagined as a result of the uncompetitive costs of peptide production and also impaired membrane selectivity of peptides between bacteria and eukaryotic cells. Although a wide variety of compounds are currently available for the treatment of cancer, there is still hope to discover a cancer application for potent cytotoxic peptides derived from bees and wasps. Our targeted pro-cytotoxic system based on mitoparan, which transports this potent cytotoxic peptide to the tumor and allows its accumulation in a controlled manner, emerges as a plausible approach to overcome off-target side effects of current cancer treatment [174]. Another promising and feasible idea tested *in vivo* to combat cancer has been presented in the form of a patent, which discloses an ultra-small lipid nanoparticle carrying melittin with potential use in clinical practice [91]. We consider that the remaining potential applications described here are currently in earlier stages of investigation and their conversion into realistic therapeutic or biotechnological applications is still far away. The future therapeutic applications of wasp and bee venom components are further complicated by strong competition with millions of potential new molecules and systems that are coming to light.
